# The Relationship of Competitive Cognitive Anxiety and Motor Performance: Testing the Moderating Effects of Goal Orientations and Self-Efficacy Among Chinese Collegiate Basketball Players

**DOI:** 10.3389/fpsyg.2021.685649

**Published:** 2021-06-01

**Authors:** Fan Peng, Li-Wei Zhang

**Affiliations:** School of Psychology, Beijing Sport University, Beijing, China

**Keywords:** competitive cognitive anxiety, motor performance, goal orientations, self-efficacy, goal profiles, moderating effect

## Abstract

The purpose of this study was to examine the moderating effects of goal orientations and self-efficacy between competitive cognitive anxiety and motor performance under conditions featuring different levels of ego-threat. Eighty-one (40 females) collegiate-level basketball players (*M* age = 20.26 years and *SD* = 2.68) completed Sport Competitive Anxiety Test, Ego Orientation in Sport Questionnaire, and General Self-Efficacy Scale prior to the experiment. Athletes participated in two sessions of free-throw tasks. After the first session, which was under a control condition, participants performed in a free-throw competitive session while being provided opponents’ scores that induced different levels of competitive cognitive anxiety. Performance is defined as the accuracy (%) in two free-throw sessions. A hierarchical multiple regression showed that high level of task-orientation and low level of ego-orientation can buffer the impairment of competitive cognitive anxiety on motor performance. The relationship between competitive cognitive anxiety and motor performance did not vary with self-efficacy. An a repeated-measured analysis of covariance after cluster analysis revealed that a high-task/low-ego profile benefited athletes the most regarding the impairment of competitive cognitive anxiety. Together, ego- and task-orientations and “goal profile” moderate the relationship between competitive cognitive anxiety and motor performance; however, self-efficacy may not serve as a moderator variable in between.

## Introduction

Mathew Emmons, a world record holder in shooting, encountered his first Olympic-sized mishap at Athens in 2004. He was leading the smallbore rifle, and a mediocre score on his final shot would have guaranteed him the gold medal. However, he fired, shooting at the bull’s eye of the target in the next lane, and received no score. His second stumble came at Beijing in 2008. Again, he had a large lead heading into the final shot, and a score of 6.7 would have been enough for him to win the gold. Unexpectedly, he hit the trigger while lowering the gun, and a score of 4.4 put him in fourth place, leaving him off the podium.

The case of Chinese shooter Du Li is different. At Athens in 2004, in the women’s 10-m air rifle event, she constantly lagged behind her main rival, Russian sharpshooter Lioubov Galkina, even before the final shot came. Surprisingly, Du got a 10.6 in the last shot, surpassed her rival Galkina, and won the gold medal.

These stories show us that competitive anxiety often interferes with motor performance. This phenomenon, often called “choking under pressure,” is a common occurrence during competitions. However, the opposite is also possible: “choking under pressure” may not happen at all, or an increase in performance under pressure, known as a “clutch performance,” may take place. It is not novel to raise the argument that competitive anxiety is not always negative and detrimental to performance (Fletcher and Hanton, 2001). Several models and theories about the mechanisms underlying the relationship between anxiety and performance have been proposed, including multidimensional anxiety theory (Martens et al., 1990a), reversal theory (Kerr, 1990), anxiety direction theory (Jones and Swain, 1992), zones of optimal functioning models (Hanin, 1980, 1986). Jones and Swain (1992), for instance, introduced the notion of “direction,” expanding the original “intense” structure of anxiety based on multidimensional anxiety theory (Martens et al., 1990a; Jones, 1995). He proposed that individuals’ interpretations of anxiety symptoms as either facilitating or debilitating to individuals affect their performance. These theories inspired many studies of the relationship between competitive anxiety and motor performance, in order to explore different ways in which diverse components of competitive anxiety can influence motor performance. In multidimensional anxiety theory, Martens et al. (1990a) divided competitive anxiety into three components: cognitive anxiety, somatic anxiety and self-confidence. Cognitive anxiety, also called “worry,” is defined as “negative expectations and cognitive concerns about oneself, the situation at hand, and potential consequences” (Morris et al., 1981, p. 541). Competitive cognitive anxiety is typically seen as negatively associated with motor performance, as it represents the degree to which individuals sense threat when evaluating the probability of achieving a desired result in a competition (Martens et al., 1990b). In other words, individuals feel competitive cognitive anxiety when they negatively evaluate the resources available for winning in a certain situation. Given the intrusive nature of competitive cognitive anxiety, it is not surprising to see many studies indicating that higher levels of competitive cognitive anxiety result in poorer performance (e.g., Chapman et al., 1997; Kurimay et al., 2017). However, a meta-analysis (Woodman and Hardy, 2003) has shown that 40% of the included studies do not support the impairment of competitive cognitive anxiety, and 23% of those have revealed an opposite result. Another meta-analysis produced in the same year (Craft et al., 2003) has also demonstrated that, on average, the relationship between competitive cognitive anxiety and performance is actually weak (*r* = 0.01, CI = [−0.03, 0.04]). The disagreement in findings with regard to the relationship between cognitive anxiety and performance was the inspiration for this study. The most straightforward way of delving into these results is moderation testing. Several moderator variables have been found for the competitive cognitive anxiety-motor performance relationship. Gender is one such variable, as a significantly greater mean effect size has been demonstrated for men (*r* = −0.22) than for women (*r* = −0.03). Other variables that have yielded similar results include standard of competition (high, *r* = −0.27; low, *r* = −0.06), type of sport (team, *r* = 0.09; individual, *r* = 0.16), and type of skill (open, *r* = 0.23; closed, *r* = 0.01; Craft et al., 2003; Woodman and Hardy, 2003). In the same vein, this study aims to test possible moderator variables that have not been adequately studied before.

Achievement goal orientations represent how individuals define success in achievement settings, and in athletic competitions, success can be defined either as mastering a skill or as indicating superior performance to others (Nicholls, 1984). These two ways of conceiving success construct different achievement motivations, labeled as “ego-orientation” and “task-orientation.” Task-orientation refers to a motivational propensity or state characterized by approaching goals, and ego-orientation refers to one characterized by avoiding goals (Nicholls, 1989). Along with achievement goal theory (Nicholls, 1984, 1989), subsequent research (Duda, 1989; Duda and Nicholls, 1992) has shown that task-orientation is related to a tendency of exerting consistent effort or persistence, as well as cooperating with others to try to fulfill the mastery of knowledge or a skill, while ego-orientation is related to the desire to attain a higher social status or other measure of superiority by outperforming others (Harwood et al., 2006). Differentiating between the two orientations is important because task-orientation tends to be positively associated with adaptive correlates and negatively associated with maladaptive correlates in sport, while ego-orientation tends to be positively associated with both maladaptive and adaptive correlates in sport (Lochbaum et al., 2016). Meanwhile, given that goal orientations are orthogonal, there are multiple ways of combination based on different levels of each goal orientation – high-task/low-ego; low-task/high-ego; or low-task/low-ego (Harwood et al., 2006) – that allow us to explore the relationships between “goal profiles” (Fox et al., 1994; Hodge and Petlichkoff, 2000) and performance in more complicated situations. It has been indicated that the balance between athletes’ goal orientations (task and ego orientations) are more important for the formation of flow experience rather than the separate level of goal orientation (Stavrou et al., 2015). The complexity of competitive situations is that situational factors such as competitiveness sometimes change how propositional goal orientations affect performance (Harwood et al., 2006). For example, an individual with a high-level of ego-orientation would probably not act as usual when in a non-competitive situation (Harwood et al., 2006). Similar insights have been witnessed in Theory of Challenges and Threat States in Athletes, which assumes that individuals tend to adopt an adaptive goal orientation when they perceive the competition as a challenge, while tend to act in the opposite manner when perceiving the competition as a threat (Jones et al., 2009). Since competitive situations influence the demonstration of goal orientations, we can assume that different levels of potential competitive threat may interact with an individual’s propensity goal orientations, as a result, may have different effects on performance. Thus, we were interested in examining the moderating effects of goal orientations in competitive settings that involve different levels of potential threat, and in exploring whether optimal “goal profiles” differ in such situations.

One of the most common potential threats in athletic competitions is the score gap between a competitor and his/her rival, which is highly related to the perception of winning/losing possibilities. Dunn and Syrotuik (2003) have identified sources of competitive anxiety as worry about failure, negative social evaluation and situational uncertainty; perceptions of score gaps are directly associated with the category of “worry about failure.” At the same time, the classification of anxiety sources hints at the possible influence of different sources of anxiety in competitive situations on the inconsistent pattern of findings across these various studies of relationships between cognitive anxiety and performance in sport. These sources are significant because of their exploration of the effects of situational factors on performance and of their potential interactive effects with individuals’ propensity to succeed or fail. Moreover, score gaps as salient potential threats are ego-threat/ego-boost situations (Vytal et al., 2013) which are potentially related to ego-orientation, and thus are potential influences on both adaptive and maladaptive correlates. Therefore, this study used score gaps as anxiety situations to test the moderating effects of goal orientations and self-efficacy between competitive cognitive anxiety and motor performance.

Self-efficacy was included in this study because it is generally regarded as a positive and facilitative factor in sport (Moritz et al., 2000; Jackson et al., 2007). The concept of self-efficacy, proposed by Bandura (1977), refers to one’s appraisal of his/her ability to obtain a certain goal via his/her actions. Individuals who have higher degrees of self−efficacy are likely to be more motivated to perform a task, and to exert greater amounts of effort and persistence (Bandura, 1997), while individuals with low self-efficacy tend to evaluate the competitive situation as more of a threat (Zilka et al., 2019). Self-efficacy is also a key predictor of performance in both physical and cognitive tasks (Feltz and Magyar, 2006). Apart from the self-appraisal of ability, one’s optimistic belief in his/her ability when experiencing frustration, which has been called “resiliency self-efficiency” (Bandura, 1997; Shipherd, 2019), is also an important factor. For example, a study on firefighters has demonstrated the moderating effect of self-efficacy on the relationship between perceived stress and burnout, which directly influences the job performance (Makara-Studzinska et al., 2019). Similarly, self-efficacy also serves as a moderating variable in the relationship between academic performance and cheating, because higher-achieving students with low levels of academic self-efficacy are more likely to cheat (Finn and Frone, 2010). As self-efficacy is considered a crucial mechanism of self-regulation (Makara-Studzinska et al., 2019), the effects of self-efficacy cannot be neglected in athletic competitions that exert tremendous pressure on their participants. However, there have been relatively few examinations of the moderation effect on self-efficacy in the relationship between competitive cognitive anxiety and motor performance.

The primary objective of this study is to address the question of whether the influence of competitive cognitive anxiety that induced by score gaps on motor performance is moderated by athletes’ achievement goal orientations and self-efficacy. In sport, given that task-orientation is regarded as facilitative, while ego-orientation could be either facilitative or debilitative (Lochbaum et al., 2016), we anticipated that a high level of task-orientation will buffer the impairment of high competitive cognitive anxiety condition that features higher levels of ego-threat (Schoofs et al., 2008) on motor performance, while a low level of ego-orientation will buffer this impairment considering our situational settings are ego-related. Apart from the individual effects of goal orientations, we also anticipated moderating effects with the same directions in respect of goal profiles. In addition, given that high self-efficacy is typically related to adaptive emotions and behaviors in sport (see Bandura, 1997), we anticipated that self-efficacy had a moderating effect in the relationship between competitive cognitive anxiety and motor performance. To sum up, we hypothesized that: (1) a high level of task-orientation and a high level of self-efficacy will buffer the impairment of high competitive cognitive anxiety on motor performance, compared to low levels of them, meanwhile, a low level of ego-orientation will buffer this impairment compared to a high level of it; (2) high-task orientation profiles (e.g., high-task/high-ego, high-task/low-ego) benefit athletes more than low-task orientation profiles (e.g., low-task/high-ego, low-task/low-ego) on motor performance regarding the impairment of competitive cognitive anxiety; and (3) low-ego orientation profiles (e.g., high-task/low-ego, low-task/low-ego) benefit athletes more than high-ego orientation profiles (e.g., high-task/high-ego, low-task/high-ego) on motor performance regarding the impairment of competitive cognitive anxiety.

## Materials and Methods

### Participants

Eighty-one Chinese collegiate-level basketball players (40 females, 41 males; *M* age = 20.26 years, *SD* = 2.68) who had won top 4 in a provincial tournament (*n* = 53: *M* age = 19.19 years, *SD* = 2.01) or participated in a national tournament (*n* = 28: *M* age = 22.52 years, *SD* = 2.54) were recruited in this study. According to G^∗^Power calculation, adopting a power of 0.8, a total sample size of 77 participants were needed for the hierarchical multiple regression and a total sample size of 72 participants were needed for the repeated-measured a repeated-measured analysis of covariance (ANCOVA). All of the participants had at least 4 years professional basketball training experience (*M* sport experience = 6.54 years, *SD* = 1.54). Three participants (2 females, 1 male) were excluded because of conscious neglecting the opponent’s scores that were provided.

### Procedures and Anxiety Conditions

After obtaining approval from the institutional research ethics board, the principal investigator contacted athletes to introduce them the study. Participants were informed that they would be required to compete a 50-free-throw competition against an opponent whose scores had been recorded and their scores would also be recorded for next participant, and they also needed to complete a few questionnaires before and after the competition. Participants were also informed to receive 30-yuan cash if won the competition. Athlete participants were voluntary to participate in the study and were treated in accordance with the ethical guidelines for human research set forth by the American Psychological Association. All participants finished written informed consent was obtained prior to commencing the experimental competition.

Data collection prior to the experimental competition was conducted via internet. The presentation order of the Sport Competitive Anxiety Test (SCAT), *Task and Ego Orientation in Sports Questionnaire* (TEOSQ), and General Self-Efficacy Scale (GSES) was counterbalanced to reduce the potential impact of any presentation order effects.

**FIGURE 1:**
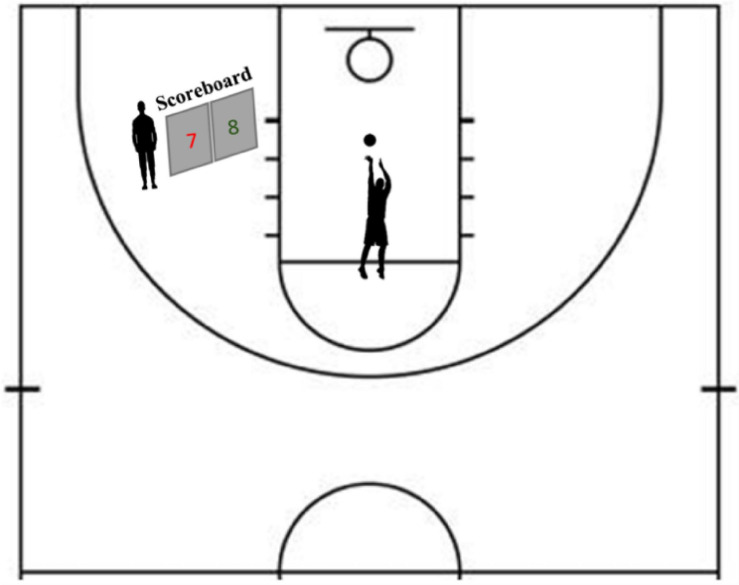
Graphical representation (not to scale) of the competition.

The experimental competition took place in half a basketball court. One athlete participated in the study at one time. He or she was required to be standing behind the free throw line to shoot (see Figure 1). Upon signing the written consent, each participant had been introduced an overview of the competition and reminded that cash was to be awarded if he or she won. Then each participant was asked to draw lots, being randomly assigned to either low level competitive cognitive anxiety (*LA*) group (*n* = 39) or high level competitive cognitive anxiety (*HA*) group (*n* = 39). After 20 throws’ practice, each participant had the first 50-free-throw without opponent but with the score recorded, followed by the first *Competitive State Anxiety Inventory-2R* (CSAI-2R) to assess participant’s anxiety during the first session (S1). Then each participant had a 50-free-throw competition with an opponent of similar ability whose scores were shown on the scoreboard after he or she had shot (see Figure 1). Participants’ scores were recorded by researchers. After that, the second CSAI-2R was filled by each athlete to assess the anxiety during the competition session (S2). Upon completion, participants completed a manipulation check items (“Have you been looking at the opponent’s scores provided during the competition?”, responses were either yes or no; “How much threat have you been feeling about that?”; responses were on a 1 = *not at all* to 7 = *very much* scale). Participants were debriefed and thanked.

Opponents’ scores were manipulated by researchers to create conditions of competitive state anxiety. There were two groups which are low competitive cognitive anxiety (*LA*) and high competitive cognitive anxiety (*HA*). For *LA* group, opponents’ scores were manipulated as lagging behind the participant and gaining 12 points (less than 25% hitting accuracy) in total, furthermore, the opponent did not gain the first point until the participant gain the first 3 points. For *HA* group, opponents’ scores were manipulated as shadowing the participant’s score during the first 40 throws, yet in the last 10 throws, opponent gained 10 points. A plot study has shown participants who were in *HA* group experienced higher intensity of competitive cognitive anxiety than those who were in *LA* group.

### Measures

Prior to coming to the experimental competition, participants completed a demographic questionnaire and modified version of the SCAT (Martens et al., 1990b), TEOSQ (Duda and Nicholls, 1992), and GSES (Schwarzer and Jerusalem, 1995) to measure competitive trait anxiety, goal orientations in sport and self-efficacy. After practice session and the experimental competition, participants were required to fill in the CSAI-2R (Cox et al., 2003), respectively.

#### Sport Competitive Anxiety Test

The SCAT (Martens et al., 1990b) contains 15 items, 10 of which (e.g., “When I compete, I worry about making mistakes”) measure symptoms associated with competitive anxiety, while the rest five are not scored for reducing the likelihood of an internal response-set bias. Items are rated on a 3-point evaluation scale including “Rarely,” “Sometimes,” or “Often” feel this way when competing in sport, with a higher score representing higher levels of competitive trait anxiety. It has been shown to have a reliable construct (internal consistency, i.e., *r* = 0.95∼0.97; test-retest reliability, i.e., *r* = 0.73∼0.88; Martens et al., 1990a), being adopted as a measure of CTA by at least 80 studies that have been published (Dunn and Causgrove Dunn, 2001). Translations of the SCAT (Zhu, 1993) into Chinese (α = 0.77) were conducted.

#### Task and Ego Orientation in Sports Questionnaire

The TEOSQ (Duda and Nicholls, 1992) contains 13 items, asking the participants to evaluate the extent of agreement when they feel most successful in a particular sport in different situations, 7 of which reflecting task-oriented (e.g., “when I learn a new skill by trying hard”), while 6 others are reflecting ego-orientation (e.g., “I feel most successful in sport when the others cannot do as well as me”). Items are rated on a 5-point Likert scale anchored between strongly disagree (1) and strongly agree (5). Translations of the TEOSQ (Chen and Si, 1998) into Chinese were conducted, which have been shown to have satisfactory internal consistencies between α = 0.71 and 0.78 (Asghar et al., 2013).

#### General Self-Efficacy Scale

The GSES (Schwarzer and Jerusalem, 1995) is an established measure of generalized self-efficacy that has been adopted in numerous studies in sport (e.g., Hewett et al., 2017; Sarı and Bayazıt, 2017). The GSES consists of 10 items (e.g., “It is easy for me to stick to my aims and accomplish my goals,” “I can solve most problems if I invest the necessary effort”) on a 4-point Likert scale, Cronbach’s alphas ranged from 0.76 to 0.90 (Schwarzer and Jerusalem, 1995). The Chinese version of the GSES (C-GSES; Zhang and Schwarzer, 1995) which has demonstrated good reliability with a Cronbach’s alpha of 0.92 were conducted (Cheung and Sun, 1999).

#### Revised Competitive State Anxiety Inventory-2R

The CSAI-2R (Cox et al., 2003) is a revised version of the Competitive State Anxiety Inventory-2 (CSAI-2; Martens et al., 1990a) that measures situational competitive anxiety in three dimensions: cognitive anxiety (5 items: e.g., “I am concerned about losing”), somatic anxiety (7 items: e.g., “My body feels tense”), and self-confident (5 items: e.g., “I’m confident about performing well”). Athletes were instructed to choose the appropriate number for each statement to indicate how they feel at this moment from 1 (not at all) to 4 (very much so). It has been revealed that CSAI-2R has stronger psychometric properties in terms of its factor structure than CSAI-2, as a Lagrange Multiplier test has shown an improved model fit after deleting 10 items from the original instrument (Cox et al., 2003). Validation studies of several versions CSAI-2R has shown adequate psychometric properties and suggested the revised version above the original, such as French (Martinent et al., 2010), Spanish (Fernández et al., 2007), Swedish (Lundqvist and Hassmén, 2005), Malaysian (Hashim and Baghepour, 2016), and Chinese (Chen et al., 2013). The Chinese version of CSAI-2R (Chen et al., 2013) were conducted. The internal consistency values for all subscales were acceptable (αs > 0.75).

## Results

### Preliminary Data Analysis

The final sample contained 40 male and 38 female participants. To maximize the sample size for data analyses, we combined data across gender into a single data set. A non-significant Box’s *M* statistic was obtained (*Box’s M* = 13.947, *F* [10, 26936.595] = 1.315, and *p* = 0.215) indicating that there were no concerns regarding the heterogeneity of variance in the two gender data sets.

Table 1 contains the descriptive statistics (i.e., means, standard deviations, and bivariate correlations [*r*]) for goal orientations, self-efficacy, S1 free-throw accuracy, and S2 free-throw accuracy. The internal consistency values for ego (α = 0.77), task (α = 0.74) subscales and self-efficacy scale (α = 0.86) were acceptable.

**TABLE 1:** Means, standard deviations, and bivariate correlations for goal orientations, self-efficacy, S1 free-throw performance, and S2 free-throw performance.

	Ego-orientation ^a^	Task-orientation^a^	Self-efficacy^b^	S1 free-throw accuracy ^c^	S2 free-throw accuracy^c^
Task-orientation	0.18				
Self-efficacy	0.02	0.35**			
S1 free-throw accuracy	−0.18	0.05	0.01		
S2 free-throw accuracy	−0.19	0.16	0.13	0.82**	
Mean	3.32	4.22	27.53	63.46	60.46
(SD)	(0.89)	(0.40)	(4.50)	(8.32)	(8.92)

SD, Standard Deviation.**p < 0.01. (*n* = 78).^*a*^Items measured on a 5-point scale. Subscale score = sum score/number of items.^*b*^Items measured on a 4-point scale.^*c*^The accuracy of each free-throw session (%). Higher figures represent better performance.

### Manipulation Check

To determine if the manipulation of the opponent’s scores in the experimental competition was successful in creating higher perceived competitive cognitive anxiety—as would be evident if participants reported different elevated levels of competitive cognitive anxiety in *HA group* than in *LA group*—ANCOVA was conducted to examine differences in competitive cognitive anxiety changing from S1 (i.e., no score manipulation) to S2 (i.e., scores comparison provided) after adjusting the means of the SCAT in different groups. Competitive trait self-control served as covariate. A statistically significant interaction was obtained: *F* (1, 75) = 58.54, *p* < 0.001, η*^2^* = 0.44, and *Power* = 1.00. More specifically, from S1 to S2, participants in *HA group* reported larger elevation in competitive cognitive anxiety intensity (*M_*S*1_* = 9.90, *SD_*S*1_* = 2.78; *M_*S*2_* = 14.03, *SD_*S*2_* = 2.76), in comparison to that of those in *LA group* (*M_*S*1_* = 10.74, *SD_*S*1_* = 3.04; *M_*S*2_* = 10.97, *SD_*S*2_* = 1.98). Manipulation check results indicated that the manipulation of competitive cognitive anxiety was successful.

### Main Analysis

#### Hierarchical Multiple Regression

**FIGURE 2:**
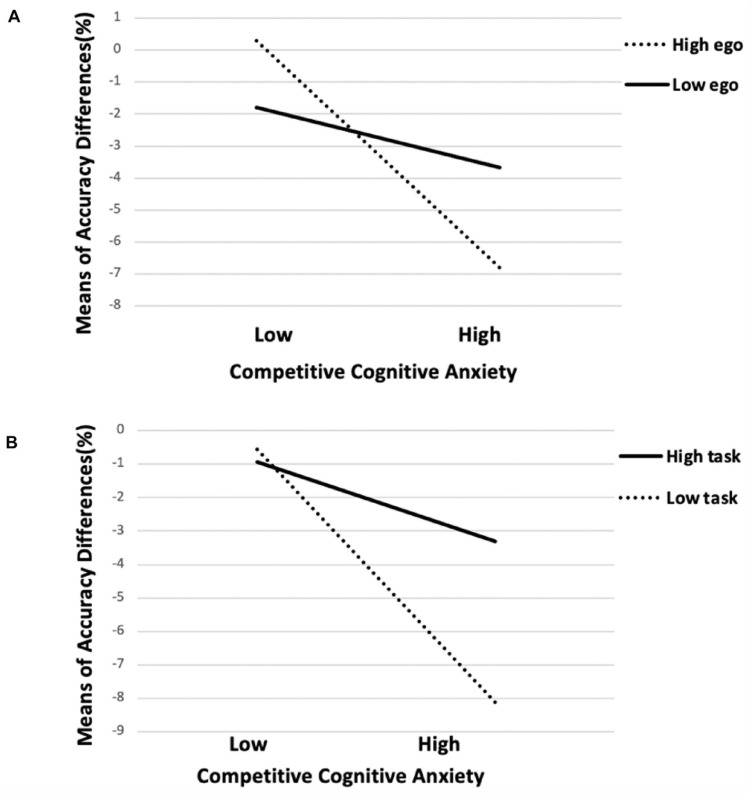
The associations of competitive cognitive anxiety with accuracy differences between two sessions (S2–S1) among athletes with high and low ego-orientations **(A)** and high and low task-orientations **(B)**. Note. High ego, high ego orientation; Low ego, low ego orientation; High task, high task orientation; and Low task, low task orientation.

Prior to conducting the regression analysis, data were screened for the presence of univariate and multivariate outliers. Subsequent data screening did not identify any univariate outliers (i.e., standardized *z*-scores for all variables ≤ | 3.29|) or multivariate outliers (i.e., all Mahalanobis distances < 18.467, *p* < 0.001: see Tabachnick and Fidell, 1996). The main dependent measure was the performance on free-throw tasks. To test hypothesis 1, we regressed S2 free-throw accuracy on S1 free-throw accuracy in Step 1 which revealed that that S1 free-throw accuracy significantly predicted S2 free-throw accuracy: *R^2^* = 0.66, *F* (1, 76) = 151.18, and *p* < 0.001. Then, we regressed S2 free-throw accuracy on participants’ ego-orientation (*M* = 3.32, *SD* = 0.89; centered), task-orientation (*M* = 4.28, *SD* = 0.39; centered), self-efficacy (*M* = 27.53, *SD* = 4.50; centered), and competitive cognitive anxiety conditions (0 = LA, 1 = HA) in Step 2, and on the bivariate interactions (ego-orientation^∗^anxiety conditions, task-orientation^∗^anxiety conditions, self-efficacy^∗^anxiety conditions) in Step 3. Competitive trait self-control (*M* = 21.33, *SD* = 3.79; centered) served as covariate. We found that adding the bivariate interactions significantly improved the predictive ability of the model, *R^2^* = 0.82, *F* (9, 68) = 38.71, *p* < 0.001, *R*^2^ change = 0.05, *F* change (3, 68) = 7.06, and *p* < 0.001. We found significant interactions in the relationship between ego-orientation and competitive cognitive anxiety (ego^∗^conditions; *B* = −1.70, *SE* = 0.52, 95% CI = [−2.72, −0.67], β = −0.24, *sr* = −0.16, and *p* = 0.002), as well as in the relationship between task-orientation and competitive cognitive anxiety (task^∗^conditions; *B* = 4.51, *SE* = 1.29, 95% CI = [0.29, 3.49], β = 0.29, *sr* = 0.17, and *p* = 0.001; see Figure 2). None of the values of tolerance was less than 0.2 and, simultaneously, all the values of VIF were less than 10, indicating no concerns related with multicollinearity. The results are shown in Table 2.

**TABLE 2:** Regression analysis predicting free-throw competition (S2) accuracy.

Predictive variables	*R*^2^	△*R*^2^	△*F*	*B*	Standardized coefficientsβ	Semi-partial correlation *sr*	*t*
Step 1	0.66		151.18***				
S1 free-throw accuracy				0.87	0.82	0.82	12.30***
Step 2	0.77	0.12	7.98***				
S1 free-throw accuracy				0.78	0.73	0.69	12.61***
Trait anxiety				0.10	0.08	0.08	1.47
Conditions				−2.79	−0.31	−0.31	−5.56***
Ego-orientation				−0.34	−0.07	−0.07	−1.23
Task-orientation				1.97	0.17	0.15	2.81**
Self-efficacy				0.06	0.06	0.06	1.05
Step 3	0.82	0.05	7.06***				
S1 free-throw accuracy				0.84	0.78	0.72	14.76***
Trait anxiety				0.13	0.11	0.11	2.17*
Conditions				−2.71	−0.31	−0.30	−6.04***
Ego-orientation				0.77	0.15	0.10	2.05
Task-orientation				−1.22	−0.11	−0.06	−1.21
Self-efficacy				0.25	0.25	0.13	2.61*
EO*conditions				−1.69	−0.24	−0.16	−3.30**
TO*conditions				4.51	0.29	0.17	3.49**
SE*conditions				−0.22	−0.17	−0.09	−1.83

Conditions, Competitive cognitive anxiety conditions; EO, Ego-orientation; TO, Task-orientation; and SE, Self-efficacy.*p < 0.05; **p < 0.01; and ***p < 0.001, all two-tailed (*n* = 78).

#### Prediction of “Goal Profiles” on Free-Throw Competition Performance

To test hypothesis 2 and 3, we conducted a cluster analysis to create goal profile groups. This method has been commonly used for classifying sample participants into groups according to their task and ego orientation scores in sport psychology (Hodge and Petlichkoff, 2000; Wang and Biddle, 2001; Cumming and Hall, 2004; Harwood et al., 2004). We employed the procedures that Harwood et al. (2004) used which combine hierarchical cluster analysis and non-hierarchical cluster analysis. Both of them offer an advantage over the traditional methods (e.g., mean- or medium-split). Instead of a formative way of classification, they provide the researcher with multiple choices of solutions that fit the data differently (Hodge and Petlichkoff, 2000). The best fit solution should reflect within-cluster homogeneity and a maximized between-cluster difference (Hair et al., 1995).

The steps were guided by the procedures outlined by Hair et al. (2006), all of the dependent measures were first standardized using z scores (*M* = 0, *SD* = l). Goal profile groups were generated through a hierarchical cluster analysis, and then were validated by a non-hierarchical cluster analysis (Harwood et al., 2004). First, for the hierarchical cluster analysis, a Ward’s method of linkage and a squared Euclidean distance were adopted to identify the number of cluster groups that should be formed by the present data. The Ward’s method was chosen for creating cluster groups because it minimizes the within-cluster variance, meanwhile, a squared Euclidean distance was chosen because it is the recommended initial distance to use when applying the Ward’s method (Aldenderfer and Blashfield, 1984). The dendogram, which is a graphical representation of all the possibilities of the classifying solutions, suggested that a 3-, 4-, or 5-cluster solution might exist in the data. However, according to the agglomeration schedule, the largest increase in the agglomeration coefficient was seen between a 4-cluster and a 3-cluster solution. Therefore, it was concluded that a 4-cluster solution best fitted the data (Hair et al., 1998). Next, A non-hierarchical cluster analysis (e.g., K-means cluster) was conducted to validate the 4-cluster solution. A 4-cluster solution was then determined to be the best fit, based on the number of participants in each cluster and similarity between the final cluster centers. We also validated the stability of a 4-cluster solution by another K-means cluster analysis with a two-thirds random sample (Hair et al., 1998). Above 92% of the sample was classified to their original clusters, confirming the stability of this 4-cluster solution. The means, standard deviations, and standardized scores for the 4 clusters are presented in Table 3.

**TABLE 3:** TEOEQ scores for clusters.

Clusters	*n*	Ego-orientation			Task-orientation		
			
		*M*	*SD*	*z*	*M*	*SD*	*z*
1. Low-ego/high-task	18	2.33	0.19	−1.11	4.59	0.20	0.78
2. Low-ego/low-task	20	2.52	0.20	−0.89	3.98	0.19	−0.77
3. High-ego/high-task	22	4.17	0.16	0.95	4.62	0.23	0.87
4. High-ego/low-task	18	4.17	0.18	0.95	3.89	0.17	−1.00

*TEOSQ, Ego Orientation in Sport Questionnaire; M, Mean; and SD, Standard Deviation.*

**FIGURE 3:**
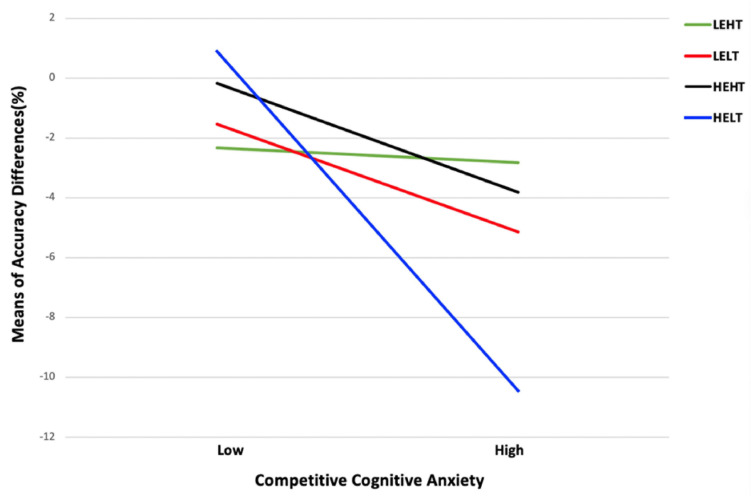
The association of competitive cognitive anxiety with accuracy differences between two sessions (S2–S1) among athletes with four goal profiles. Note. LEHT, Low-ego/high-task; LELT, low-ego/low-task; HEHT, high-ego/high-task; and HELT, high-ego/low-task.

The interpretations of goal profile groups of being as low or high on the two goal orientations was using a *z* score criterion of ±0.50 (Hodge and Petlichkoff, 2000; Wang and Biddle, 2001; Harwood et al., 2004). According to this criterion, 18 participants in Cluster 1 had low-ego/high-task profiles, 20 participants in Cluster 2 had low-ego/low-task profiles, 22 participants in Cluster 3 had high-ego/high-task profiles, and 18 participants in Cluster 4 had high-ego/low-task profiles. A MANOVA was then employed to examine whether significant differences existed between the cluster groups on their task- and ego-orientation scores. A significant multivariate effect was found for goal orientations, *Pillai’s Trace* = 1.71, *F* (6, 148) = 144.58, *p* < 0.001, η*^2^* = 0.85, and *Power* = 1.00, with an observed power of 100%. Significant univariate effects were found for ego orientation (*F* [3, 74] = 590.03, *p* < 0.001, η*^2^* = 0.96, and *Power* = 1.00) and task orientations (*F* [3, 74] = 73.12, *p* < 0.001, η*^2^* = 0.75, and *Power* = 1.00). A *post hoc* test showed that participants in Cluster 3 (high-ego/high-task) and Cluster 4 (high-ego/low-task) had a significantly higher score on ego orientation than participants in Cluster 1 (low-ego/high-task) and Cluster 2 (low-ego/low-task; *ps* < 0.01), while participants in Cluster 1 (low-ego/high-task), and Cluster 3 (high-ego/high-task) had a significantly higher score on ego orientation than participants in Cluster 2 (high-ego/low-task) and Cluster 4 (low ego/low-task; *ps* < 0.01). After labeling these groups, ANCOVA was calculated to examine for differences among the cluster groups under competitive cognitive anxiety conditions. Competitive trait anxiety served as the covariant. A significant interaction between competitive cognitive anxiety and goal profile groups, *F* (3, 69) = 5.85, *p* = 0.001, η*^2^* = 0.20, and *Power* = 0.94. Probing the interaction (see Figure 3) revealed that a high-task/low-ego profile benefited athletes the most under a high competitive cognitive anxiety condition, whereas a high-ego/low-task profile was shown to be the most detrimental to motor performance under a high competitive cognitive anxiety condition.

## Discussion

The primary purpose of this study was to examine the moderating effects of goal orientations and self-efficacy on the relationship between competitive cognitive anxiety and motor performance. Overall, our findings indicate that ego- and task-orientations and “goal profiles” moderate the relationship between competitive cognitive anxiety and motor performance; however, self-efficacy may not serve as a moderating variable.

Current research sheds important light upon relationships between competitive cognitive anxiety and motor performance. Previous investigations focused more on the main effects of goal orientations, goal profiles and self-efficacy on motor performance. Although those findings indicated the significant influences of these variables on athletic performance, a greater understanding of their moderating effects under stress will be more beneficial to researchers and practitioners in sport. This study examined the moderating effects of goal orientations, goal profiles and self-efficacy separately via multiple methods of analysis.

Previous studies have noted that in sport, task-orientation tends to be positively associated with adaptive correlates, while ego-orientation tends to be positively associated with both maladaptive and adaptive correlates (Lochbaum et al., 2016). The interaction effects we found in the current study extend previous research by illustrating the influence of goal-orientations on athletic performance under a high competitive cognitive anxiety. In this study, hierarchical multiple regression results (Table 2) revealed that higher task-orientation tends to benefit individuals more than lower task-orientation (β = 0.29, *p* = 0.001), while lower ego-orientation tends to benefit individuals more than higher ego-orientation (β = −0.24, *p* = 0.002), in terms of free-throw accuracy, under a high competitive cognitive anxiety condition that contains scores indicating lagging behind. In other words, these results showed that the contributions of high task-orientation and low ego-orientation were reflected not only in directly improving athletes’ motor performance, but also in building “a facilitative process” intended to help athletes cope with high competitive cognitive anxiety. These results are in line with the findings of Albert et al. (2021) who proposed that task-orientation has positive correlations with multiple significant predictors of performance under stress, such as grit (Albert et al., 2021), and self-confidence (Lee et al., 2020). Importantly, in the current study we identify a dispositional characteristic that can buffer the impairment of competitive cognitive anxiety. In this regard, this study provided another possible explanation for the inconsistent findings concerning the relationship between competitive cognitive anxiety and motor performance. According to our findings, ego- and task-orientation both serve as moderator variables in this relationship. Regardless of the underlying reasons that explain why task orientation may boost athletic performance, the current findings strengthen Turner et al.’s (2021) findings and reveal that challenges and threat evaluations predict the performance under pressure (Turner et al., 2021), given that athletes tend to adopt task-orientation when they evaluate the competition as a challenge. With regard to the reverse moderating effect of ego orientation, Jones et al. (2009) has proposed that when athletes perceive the competition as a threat, they may be more likely to adopt maladaptive goal orientations. Therefore, it is possible that in the ego-threat situation we created based on the score gaps, athletes with higher levels of ego-orientation are more prone to act maladaptively than those with lower levels (Kavussanu et al., 2014).

In the same hierarchical multiple regression analysis, we also examined the moderating effect of self-efficacy. Contrary to our hypothesis, our findings did not show a statistically significant interaction between self-efficacy and competitive cognitive anxiety conditions (β = −0.17, *p* = 0.07). One possible interpretation for this result is that high and low self-efficacy may have an equal influence on the relationship between competitive cognitive anxiety and motor performance. Moreover, the main effect of self-efficacy that was regressed in the Step 2 was not significant as well (β = −0.06, *p* = 0.30). It also implied that in comparing a free-throw competition session that combines high and low competitive cognitive anxiety conditions to a non-competition session, we failed to witness significantly different effects of self-efficacy on free-throw accuracy. Our results demonstrated that self-efficacy affected motor performance under both competitive and non-competitive conditions equally. This result, therefore, is not inconsistent with previous findings showing a positive association between self-efficacy and motor performance (e.g., Sklett et al., 2018). It is worth mentioning that this method of analysis allowed us to examine “purer” moderating effects by accounting for the non-competition performance in the first step, which contains irrelevant factors that potentially interact with moderator variables that predict motor performance. It also emphasized our objective of testing moderating effects on the relationship between competitive cognitive anxiety and motor performance which indicated a distinct feature of “competitiveness.” Thus, the focus of our study was the moderating effects in our hierarchical multiple regression, rather than the main effects.

Apart from investigating two independent moderating effects of goal orientations, we also attempted to examine the moderating effect of “goal profiles,” which present the combination of both goal orientations, between competitive cognitive anxiety and motor performance. We first followed the procedures described by Harwood et al. (2004) to group the sample athletes into different profiles, in which a hierarchical and a non-hierarchical cluster analysis were successively conducted. Four cluster groups emerged from the analysis, which we labeled as low-ego/high-task (cluster 1), low-ego/low-task (cluster 2), high-ego/high-task (cluster 3), and high-ego/low-task (cluster 4) according to a *z*-score criterion of +0.5 (Hodge and Petlichkoff, 2000; Wang and Biddle, 2001). We then conducted an ANCOVA, the result of which supported our hypothesis that a profile consisting of a low ego orientation and a high task orientation benefits athlete the most when they are under a high competitive cognitive anxiety condition. This result is in keeping with what we have found in hierarchical multiple regression analysis, that the interaction effect demonstrates that the “low-ego/high-task” profile benefits athletes the most under a high competitive cognitive anxiety condition. In addition, ANCOVA results extend the regression results by revealing that goal orientations can be complementary under our experimental settings. More specifically, the “high-ego/high-task” profile and the “low-ego/low-task” seem to indistinctly affect athletes’ motor performance under competitive cognitive anxiety conditions (see Figure 3). In comparing our findings to a previous study that has examined the relationship between goal profiles and psychological skills use (i.e., Harwood et al., 2004), we note some differences. Unlike Harwood et al. (2004) which has concluded that young athletes with “moderate-ego/high-task” tend to apply more psychological skills during competitions, we identified that under a high competitive cognitive anxiety the most performance boosting goal profile is “low-ego/high-task.” In explaining this difference, it is possible that labels are sample-specific so conclusions drawn from multiple cluster patterns can be different. Although the limitation of the cluster analysis in result generalizing seems salient, the unique contribution of this analysis is to expand our knowledge regarding the balance of goal orientations which serve as two orthogonal dispositional characteristics (Sicilia et al., 2008; Harwood et al., 2015).

### Practical Implications

Our findings have some important practical implications. First, we hope that practitioners in sport psychology will pay more attention to the applied importance of goal achievements in optimizing athletic performance. We propose that sport psychologists and coaches focus on helping athletes reduce ego-orientation and improve task-orientation. Athletes with high levels of ego-orientation tend to perform worse when experiencing high levels of competitive cognitive anxiety, and those with high levels of task-orientation seem to have more adaptive regulation in a situation that contains significant competitive threats. Focusing part of the mental training program before competition on goal orientations is likely to enhance athletic performance (Hogue et al., 2013; Hogue, 2020). Consequently, developing and implementing proper motivational climate interventions that specifically target the reduction of ego-orientation and the promotion of task-orientation seem essential, especially for younger athletes (Harwood et al., 2004). In addition, we suggest that in the face of competitive threat, a high level of task orientation and/or a low level of ego orientation may buffer the impairment of athletic performance due to anxiety. Thus, developing pre- and within-competition routines based on fostering a beneficial goal profile that may help athletes keep the necessary focus in front of competitive threat can be a great asset in an unpredictable competition. In light of our findings, we highly recommend developing interventions to shape optimal goal orientations across stages of preparation, pre- and within-competition.

### Limitations and Future Directions

Certain limitations of our study should not be overlooked. First, caution must be applied when generalizing the results of this study that are related to cluster analysis. The labels in each cluster group are created in relation to the z scores and are therefore sample-specific. Even though the unstandardized means difference between a lower (*M* = 2.43) to a higher (*M* = 4.17) ego orientation and that between a lower (*M* = 3.94) and higher (*M* = 4.61) task orientation seem fair, we can easily recognize a typical positive skewing of task orientation (Harwood et al., 2004). It seems more noteworthy when we attempt to emphasize the balance of both goal orientations; yet the criteria for labeling them are not identical. Thus, although evidence supported the effectiveness of our cluster solution grouping the participants distinctly, we should be meticulous when generalizing these findings to a wider athletic population. Apart from that, as the sample participants in current study were limited to Chinese athletes, caution must also be applied when generalizing these findings to multi-ethnic athletes.

With regard to future work, researchers should consider exploring the neuroscientific mechanism that leads to debilitative outcomes among individuals with high levels of ego-orientation. In other words, determining the exact process of high level of ego-orientation impairing motor performance under a high competitive cognitive anxiety condition is imperative. More research is also needed to explore additional potential mediators, such as interpretations of anxiety and self-control.

## Conclusion

The results of this study highlight the benefit of task-orientation when athletes face competition consisting of ego-threat conditions. The results also reinforce the importance of reducing ego orientation as much as possible for better preparation, as well as a situational coping strategy for the competition. Finally, the results do not support the moderating effect of self-efficacy, which indicates that self-efficiency may not have special effects on motor performance under a competitive condition when the level of cognitive anxiety seems high.

## Data Availability Statement

The raw data supporting the conclusions of this article will be made available by the authors, without undue reservation.

## Ethics Statement

The studies involving human participants were reviewed and approved by Psychology Ethics Committee, School of Psychology, Beijing Sport University. The patients/participants provided their written informed consent to participate in this study.

## Author Contributions

FP and L-WZ conceived the study. FP collected data. FP planned the analytical approach, performed the analyses, and wrote the manuscript, with feedback from L-WZ. Both authors approved the content of the manuscript.

## Conflict of Interest

The authors declare that the research was conducted in the absence of any commercial or financial relationships that could be construed as a potential conflict of interest.
